# Isotopic composition and source of plutonium in the Qinghai-Tibet Plateau frozen soils

**DOI:** 10.1038/s41598-019-44391-0

**Published:** 2019-05-27

**Authors:** Junwen Wu

**Affiliations:** 1 Institute of Marine Biology, College of Science, Shantou University, Shantou, 515063 China; 2 State Key Laboratory of Marine Environmental Science, Xiamen University, 361102 Xiamen, China

**Keywords:** Environmental chemistry, Environmental impact

## Abstract

The ^239+240^Pu activities and ^240^Pu/^239^Pu atom ratios in the frozen soils of the Yellow River Source Area (YRSA) were determined to examine the Pu source and evaluate its environmental risk. The ^239+240^Pu activities of surface frozen soils in the YRSA, ranging from 0.053 to 0.836 mBq g^−1^, are comparable to those observed in China elsewhere (0.005–1.990 mBq g^−1^). The ^240^Pu/^239^Pu atom ratios of surface soils in the YRSA are in the range of 0.168–0.201 (average = 0.187 ± 0.012, n = 6), comparable to the global fallout of 0.180 ± 0.014. Based on the latitudinal and spatial distribution of Pu isotopic composition, I clarified that the Pu source is mainly from global fallout at present. The activity levels of Pu in the YRSA do far not cause a Pu toxicity to the downstream drinking water even the frozen soil begins to melt and release Pu to the Yellow River. However, since close-in fallout from Lop Nor where the Chinese nuclear tests were carried out during 1964–1980, high deposition and accumulation of Pu was observed in the Chinese soil cores through synthesizing an expanded Pu dataset, which alerts us it is necessary to further monitor the Pu activity levels in the YRSA soil cores to ensure the safety of downstream drinking water. Finally, I point out that information on Pu isotopes would help in establishing a baseline for future environmental risk assessment.

## Introduction

Plutonium (Pu), a transuranic element, was synthesized for the first time in 1940s^1^. Since then, it has been largely introduced into the environment as a result of global atmospheric nuclear weapons testing^2,3^ and reprocessing plants of nuclear materials^4,5^ and various accidental releases (*e.g*., Chernobyl and Fukushima)^6,7^. The two major Pu isotopes, ^239^Pu (half-life, t_1/2_ = 24110 years) and ^240^Pu (t_1/2_ = 6524 years), both alpha-emitters, in the water and air of populated areas affected by the Pu sources, have been of great environmental and societal concerns due to high toxicity, long half-lives and the exposure risk of internal radiation. It was reported that a total of 543 atmospheric nuclear weapons testing released ~11 PBq (1 PBq = 10^15^ Bq) ^239+240^Pu into the earth environment during the period of 1945–1980^8^. When deposited in marine environment or water system, Pu participates in a variety of environmental processes and can be transported rather far from the source point. Such environmental concerns can thus be beyond a regional scale because of this transport. For example, Pu isotopes of sediment and seawater in the China Sea suggested such transport from close-in fallout from the Pacific Proving Grounds in the Marshall Islands where a large-scale USA nuclear testing was carried out in 1950s^9,10^. Nevertheless, in terrestrial environment or soil, Pu is strongly associated to the fine particles and the organic matter^11^, so is not easy to transport far away while settling down.

The ^240^Pu/^239^Pu atom ratio is widely applied to trace the Pu source because Pu isotopic ratios vary with reactor type, nuclear fuel burn-up time, neutron flux and energy, and for fallout from nuclear detonations, weapon type and yield^9^. The average ^240^Pu/^239^Pu atom ratio of global fallout is characterized as 0.180 ± 0.014^12^. Reactor-grade Pu has higher ^240^Pu/^239^Pu atom ratios ranging from 0.2 to 1.0 depending on the fuel burn-up, for example, the ^240^Pu/^239^Pu atom ratios of Chernobyl and Fukushima accidents were characterized by 0.38 ± 0.07^13–15^ and 0.30–0.38^7,16^, respectively. Weapons-grade Pu has a lower ^240^Pu/^239^Pu atom ratio (0.01–0.07)^17,18^. Over the past few decades, the Pu isotopes have been extensively investigated in order to elucidate their source terms and to assess their environmental impact and behavior^19,20^.

The Yellow River Source Area (YRSA), located in the northeast edge of the Qinghai-Tibet Plateau, is characterized by permafrost and high elevation (>4000 m)^21^. The YRSA includes over 4000 lakes with a total water surface of 1664.6 km^2^, 48 of which have a water surface larger than 0.5 km^2^. The two largest freshwater lakes in the YRSA are Ngoring Lake (34.767°~35.083°N, 97.533°~98.900°E) with a surface area of ~610 km^2^ and a total water volume of 10.8 × 10^9^ m^3^, and Gyaring Lake (34.817°~35.001°N, 97.050°~98.450°E) with a surface area of ~525 km^2^ and a total water volume of 4.8 × 10^9^ m^3^. The YRSA is the river’s major source area of streamflow and is also called “water tower” of the Yellow River. The Yellow River, the second largest river of China, has a catchment area of >750 000 km^2^ and 5464 km length^22^, which flows from north-west to south-east with an average discharge of 22.6 m^3^ s^−1^ recorded by the Huanghe (Yellow River) hydrological station (34.885°N, 98.172°E) during 1955–2005. The Yellow River, called the “mother river” of China, is critical to water resource and security of China. Meanwhile, the YRSA is located the downwind area from the Chinese nuclear tests in Lop Nor where 22 atmospheric nuclear tests were carried out during the period of 1964–1980^8^ (Fig. S1 in the supplementary information). Thereinto, the six large nuclear tests of Lop Nor released a great number of radioactive debris (~18.5 Mt). The man-made radionuclides rather than just global fallout occurred in this area as local fallout. Therefore, the potential impact of artificial radionuclides and radioactive source identification in the YRSA is an issue of great concern to the public. To date, however, there has no Pu data reported for the YRSA frozen soil. This study, for the first time, reported the Pu isotopic ratio and activity level of frozen soil in the YRSA and sought to examine Pu source. Such source identification is further aided by the measurements of the relative abundance of Pu isotopes. For this study, I also aimed at investigating spatial distribution of Pu in the soil of China by synthesizing an expanded Pu dataset. Finally, I contend that information on Pu activities and isotopic ratios would greatly help in establishing a baseline for future environmental radiological risk assessment.

## Results and Discussion

### Isotopic composition and source of Pu in the YRSA

#### ^240^Pu/^239^Pu atom ratio

Pu in soil tends to partition to special particles rather than to sorb homogeneously to all particles^23^. In order to avoid the effect of particle size, the soil samples (<63 µm) have been homogenized and are primarily composed of clay.

The ^240^Pu/^239^Pu atom ratios of surface soils in the YRSA ranged from 0.168 to 0.201 (average = 0.187 ± 0.012, n = 6), which were comparable to those observed in the Qinghai Lake (0.172–0.221, average = 0.189 ± 0.028, n = 3)^24^, at the distance of about 300 km from our studied area. Within the distance of 400 km from our investigated area, the ^240^Pu/^239^Pu atom ratios in the Sugan and Shuangta Lakes located in the downwind of Lop Nor were reported to be 0.166–0.188, with an average of 0.177 ± 0.011 (n = 3)^25^. At the northwest (upwind) 250 km of Lop Nor, the ^240^Pu/^239^Pu atom ratios in the Bosten Lake were observed to be 0.168–0.184, with a mean of 0.178 ± 0.012 (n = 3)^26^. Therefore, the surface ^240^Pu/^239^Pu atom ratios of soils/sediments around the YRSA are comparable to that of global fallout. This implies that: (1) the Pu in this area is mainly sourced from the global fallout; (2) the ^240^Pu/^239^Pu atom ratio, if there has any additional Pu source, is similar as that of global fallout. The possible additional sources in this area are consist of the nuclear accidents (e.g., Chernobyl and Fukushima), Semipalatinsk and Lop Nor nuclear tests. The ^240^Pu/^239^Pu atom ratio of Chernobyl nuclear accident was characterized by 0.408^14^ and Kim *et al*.^27^ confirmed that the Pu contribution derived from the Chernobyl nuclear accident was negligible in the marginal seas of the North Pacific Ocean. The ^240^Pu/^239^Pu atom ratio was also featured by 0.30–0.38 for the Fukushima nuclear accident^7,16^ and our previous work in the China Sea suggested that there had no Pu signature from the Fukushima nuclear accident^9^. Meanwhile, the YRSA is far away from Chernobyl and Fukushima nuclear accidents than the China Sea. The ^240^Pu/^239^Pu atom ratios (~0.18) in the Chinese profile soils reveal that the direct input of close-in fallout from the Semipalatinsk nuclear tests did not lead to any significant Pu contribution in these areas^28,29^, in view of its distinctive ^240^Pu/^239^Pu atom ratios of 0.03–0.05^30^. Therefore, we can expel the Pu signatures from the Chernobyl and Fukushima nuclear accidents, and Semipalatinsk nuclear tests. Nevertheless, the ^240^Pu/^239^Pu characteristic atom ratio of Lop Nor has not been well defined up to date due to be lack of systematic studies. Leifer and Toonkel^31^ measured high ^240^Pu/^239^Pu atom ratio (~0.224) in the atmospheric debris after the largest testing of Lop Nor (~4 Mt) on November 17, 1976. However, low ^240^Pu/^239^Pu atom ratios (0.080–0.103) in deep sediments were also observed in the downwind of Lop Nor, indicating the Pu atom ratio of Lop Nor could be lower than that of global fallout at this period^25,26^. Accordingly, Bu *et al*.^32^ inferred that the ^240^Pu/^239^Pu atom ratios of Lop Nor were possibly in the range of 0.059–0.224 based on their observation in the downwind from the Lop Nor site in the Jiuquan area and the above published Pu data^25,26,31^. Therefore, they cannot well define the ^240^Pu/^239^Pu characteristic atom ratio of Lop Nor because of the sporadic reports and/or Pu dataset. Here, I combined the most complete Pu dataset in the Chinese surface soils up to date and did a statistical analysis in order to well define the ^240^Pu/^239^Pu characteristic atom ratio of Lop Nor.

**Figure 1:**
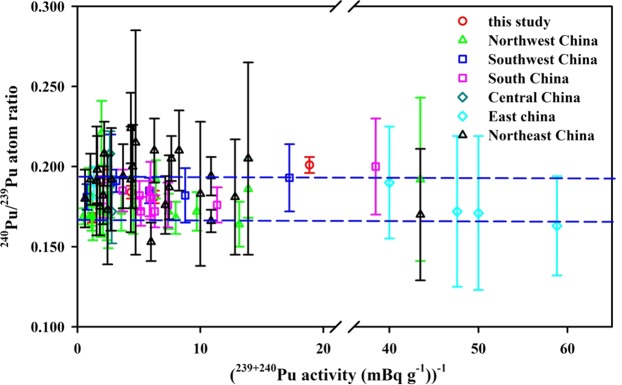
Plot showing the relationship between ^240^Pu/^239^Pu atom ratio and the reciprocal of ^239+240^Pu activity for surface soils of China. Blue dashed lines represent the average ^240^Pu/^239^Pu atom ratio (0.180 ± 0.014) of global fallout^12^. Pu data of surface soils/sediments are cited from previous studies (Northwest China^24–26,29,32^; Southwest China^29,33–35^; South China^9,36^; Central China^37^; East China^35,38^; Northeast China^39,40^).

**Figure 2:**
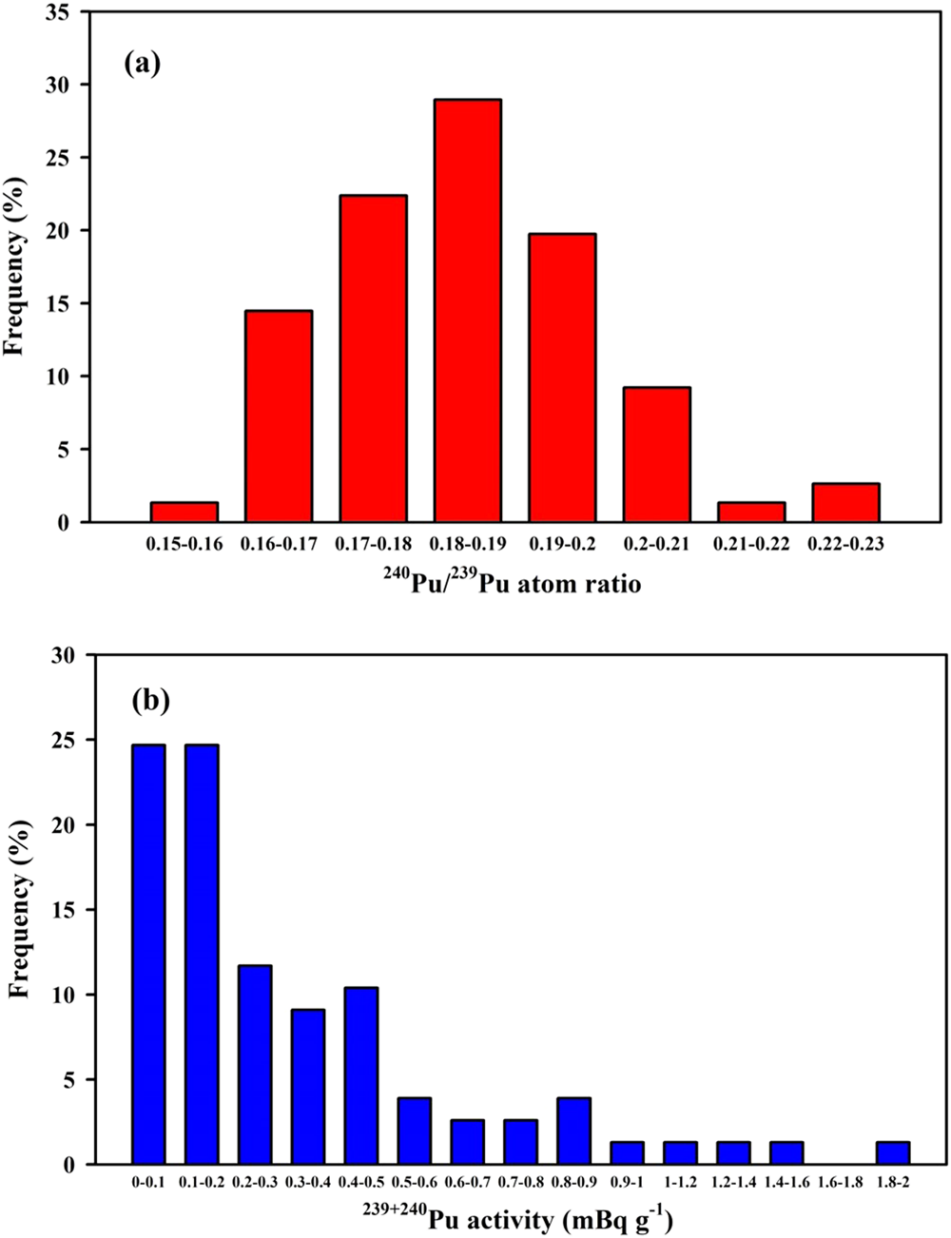
Frequency distributions of ^240^Pu/^239^Pu atom ratios (**a**) and ^239+240^Pu activities (**b**) in Chinese surface soils. Pu data sourced as same as Fig. 1.

Indeed, our ^240^Pu/^239^Pu atom ratios (0.187 ± 0.012, n = 6) were also comparable to the previous observations made elsewhere in the Chinese surface soils (0.183 ± 0.018, n = 71; Fig. 1), namely, Northwest China (0.178 ± 0.013, n = 21)^24–26,32^, Southwest China (0.187 ± 0.005, n = 6)^29,33–35^, South China (0.181 ± 0.008, n = 10)^9,36^, Central China (0.190 ± 0.025, n = 2)^37^, East China (0.177 ± 0.012, n = 5)^35,38^ and Northeast China (0.187 ± 0.023, n = 27)^39,40^. As shown in Fig. 1, the relationship between ^240^Pu/^239^Pu atom ratio and the reciprocal of ^239+240^Pu activity in the Chinese surface soils suggested the global fallout was a dominated Pu source. It is noted that the ^240^Pu/^239^Pu atom ratios have the different uncertainties in the different laboratories, which were potentially caused by the measuring instruments (e.g., MC–ICP–MS, SF–ICP–MS, AMS, TIMS). Whilst, our Pu data with the low uncertainties are mainly attributed to the MC–ICP–MS with multiple ion counting detectors (1–4) using synchronous measurements. In addition, I further analyzed the big dataset of Pu in the Chinese surface soils and plotted the frequency distribution of ^240^Pu/^239^Pu atom ratios in Fig. 2a, showing a typical Gaussian/normal distribution. Among the 77 surface soil samples, the ranges of 0.17–0.18 and 0.18–0.19 account for 22% and 29%, respectively. Approximate 86% of ^240^Pu/^239^Pu atom ratios fall within the range of global fallout, which further confirmed the major Pu source in Chinese surface soils was from the Pu deposition of global fallout. The ^240^Pu/^239^Pu atom ratios in the Chinese surface soils are in good agreement with those observed in Japan surface soils collected around 1970s (0.183 ± 0.018) where the global fallout is also the dominated Pu source as suggested by Yang *et al*.^41^. In summary, I suggest, if the Pu additional source in the surface soil from Lop Nor, that the Pu atom ratio of Lop Nor should be identical to that of global fallout (0.180 ± 0.014). Indeed, a similar case occurred in the Enewetak atoll, the low ^240^Pu/^239^Pu atom ratios in the Lujor island (0.111–0.117) and Runit island (0.062–0.070) were also observed, but the ^240^Pu/^239^Pu atom ratios in the Marshall Islands were characterized by 0.300–0.360 because the high ^240^Pu/^239^Pu atom ratios were produced from the mainly large tests^42^.

**Figure 3:**
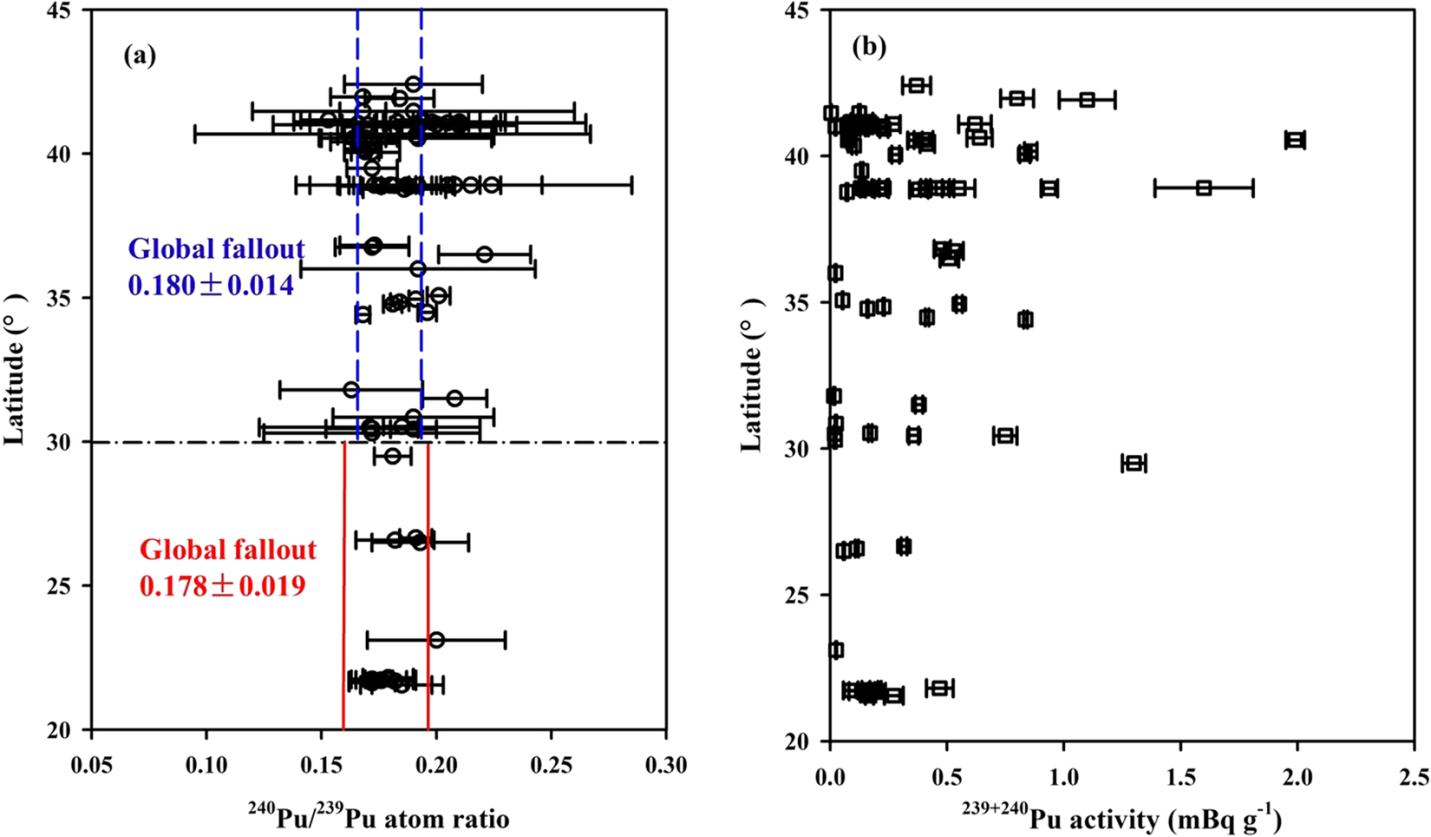
Latitudinal distributions of ^240^Pu/^239^Pu atom ratio (**a**) and ^239+240^Pu activity (**b**) in the surface soils of China. Red solid lines and blue dashed lines represent the average ^240^Pu/^239^Pu atom ratio of global fallout in the different latitudinal zone, namely, 0.178 ± 0.019 (0–30°N) and 0.180 ± 0.014 (30–70°N)^12^. Pu data sourced as same as Fig. 1.

**Figure 4:**
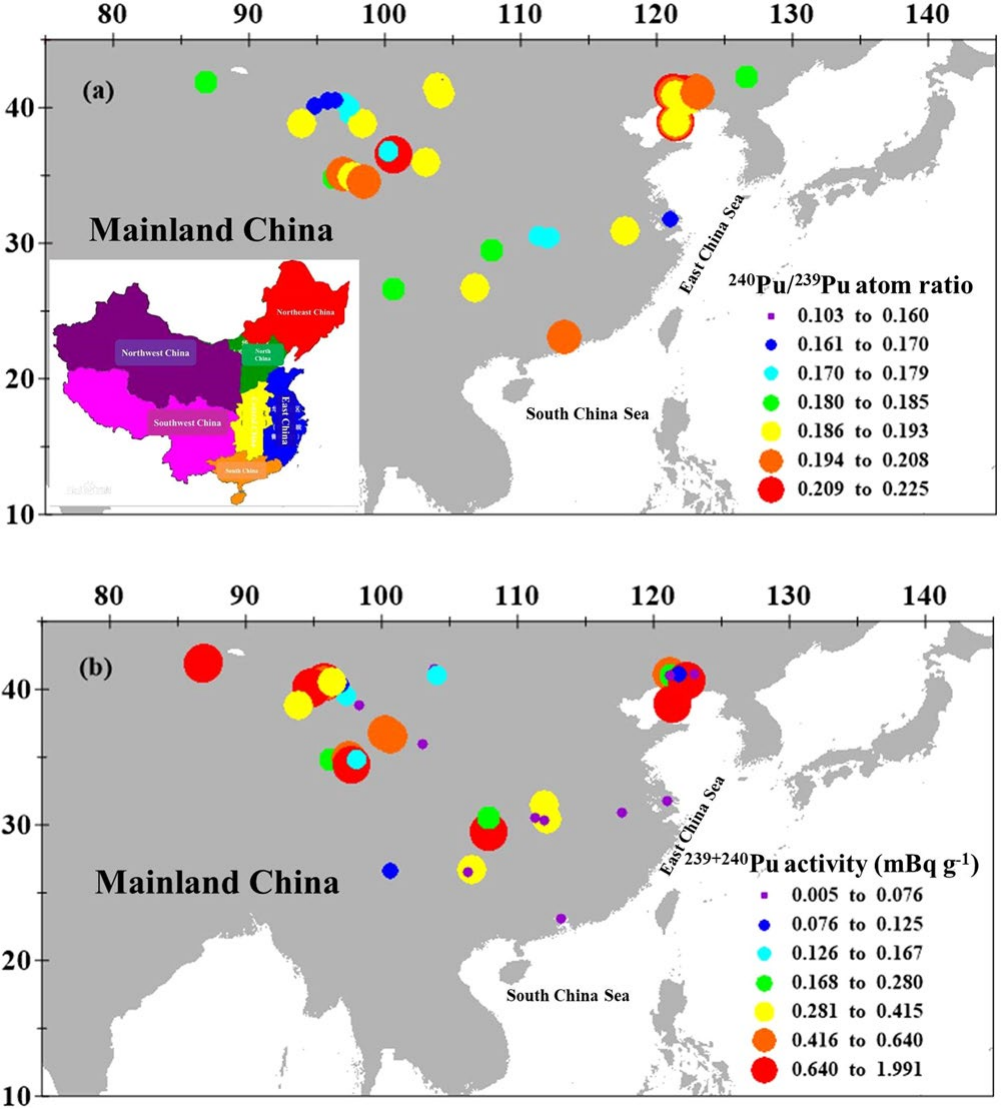
Distributions of (**a**) ^240^Pu/^239^Pu atom ratio and (**b**) ^239+240^Pu activity of the surface soil in China. Pu data of surface soils/sediments are cited from the previous studies (Northwest China^24–26,29,32^; Southwest China^29,33–35^; South China^9,36^; Central China^37^; East China^35,38^; Northeast China^39,40^). This map was prepared with surfer 10.0 software.

I also firstly presented the latitudinal distribution of ^240^Pu/^239^Pu atom ratios in the Chinese surface soils in Fig. 3a, showing a slight increase with the latitude. For example, they increased from 0.180 at the latitudinal zone of 20–30° to 0.186 at the latitudinal zone of 30–45°. Such pattern is also very consistent with the latitudinal distribution of global fallout, indicating the Pu in the surface soil of China is mainly sourced from the global fallout. Combined with the most complete Pu dataset up to now, the spatial distribution of ^240^Pu/^239^Pu atom ratio in the Chinese surface soils are also presented in Fig. 4a, showing a positive relationship with the latitude, as similar as the deposition of global fallout. Meanwhile, I did statistical analysis using a simple F–test and T–test in detail described in elsewhere^43^. The result shows the ^240^Pu/^239^Pu atom ratios between the Chinese surface soils and the global fallout had no significant difference (student t–test, p = 0.06 > 0.05). The horizontal distribution of ^240^Pu/^239^Pu atom ratio in the Chinese surface soils overall showed a uniform distribution pattern in the seven zones of China. Therefore, all this implies that the Pu of surface soils in these areas is mainly sourced from the global fallout at present. And then, I further examine the activity levels and inventories of ^239+240^Pu in the Chinese soils.

#### ^239+240^Pu activity

Frozen soils are usually formed under long-term weathering, abrasion, handling and sedimentation conditions^44^. The mechanical properties of structure in the frozen soils are different from the other soil types^45^. There has no report about Pu isotopic composition in the frozen soil up to date. Here, I present the ^239+240^Pu activities of surface frozen soils in the YRSA in Table S1, ranging from 0.053 to 0.836 mBq g^−1^, an average of 0.37 ± 0.29 mBq g^−1^ (n = 6). Such ^239+240^Pu activity levels were also comparable to those observed in the vicinity of our investigated area (within 500 km), for example, Qinghai Lake (0.48–0.53 mBq g^−1^, n = 3)^24^, Sugan and Shuangta Lakes (0.22–0.40 mBq g^−1^, n = 3)^25^. Combined with the previously published Pu dataset, I analyzed the ^239+240^Pu activities in the seven areas of China, namely, Northwest China (0.005–1.990 mBq g^−1^, an average of 0.452 ± 0.469 mBq g^−1^, n = 21)^24–26,28,32^, Southwest China (0.058–1.300 mBq g^−1^, average = 0.388 ± 0.462 mBq g^−1^, n = 6)^29,33–35^, North China (0.066–0.149 mBq g^−1^)^46^, South China (0.026–0.469 mBq g^−1^, an average of 0.188 ± 0.119 mBq g^−1^, n = 10)^9,36^, Central China (0.358–0.380 mBq g^−1^, average = 0.369 ± 0.016 mBq g^−1^, n = 2)^37^, East China (0.017–0.750 mBq g^−1^, an average of 0.167 ± 0.326 mBq g^−1^, n = 4)^35,38^ and Northeast China (0.023–1.600 mBq g^−1^, an average of 0.317 ± 0.339 mBq g^−1^, n = 27)^39,40^. The surface ^239+240^Pu activity levels in the frozen soils were comparable to those in other soil types, which is potentially related to their similar organic carbon content and grain size^29,35,39,47,48^. Overall, the ^239+240^Pu activities in Chinese surface soils, ranging from 0.005 to 1.990 mBq g^−1^, were comparable with the activity levels of Pu in soils in Japan (0.07–4.31 mBq g^−1^)^41^ and in Korea (0.24–1.1 mBq g^−1^)^49^ before the Fukushima Daiichi nuclear accident. The spatial distribution of ^239+240^Pu activities in the Chinese surface soils are also presented in Fig. 4b, showing quite heterogeneous pattern, which was potentially caused by the multifarious factors, such as the physical and chemical properties of soil (e.g., mineral composition and the contents of organic matter), bacteria activity (e.g., anaerobic sulfate-reducing bacteria), precipitation, Eh, pH, the oxidation state and ionic size of Pu isotopes^11,49–51^. In general, the ^239+240^Pu activity is positive correlation with the precipitation and the concentration of organic matter^28,29,39,49^. The Pu speciation and its partition with soil particles size were influenced by the pH of soil. It is reported that the *K*_*d*_ (distribution coefficient) of Pu in the bentonite was ~40% lower at 3 of pH than that at pH = 7 because the Pu at higher pH would help in being associated with soil particles^52^. In addition, the ^239+240^Pu activities in the Chinese surface soils also show a wider variability range at the latitudinal zone of 20–45° and their latitudinal distribution also corresponds well with the expected deposition of global fallout (Fig. 3b). For example, they increased from 0.233 mBq g^−1^ at the latitudinal zone of 20–30° to 0.382 mBq g^−1^ at the latitudinal zone of 30–45°, possibly related to the latitudinal distribution of ^239+240^Pu deposition; i.e., high fluxes occur at mid–latitudes and low fluxes occur at low–latitudes^53^.

### Vertical distribution of Pu isotopic composition in the Chinese soils

**Figure 5:**
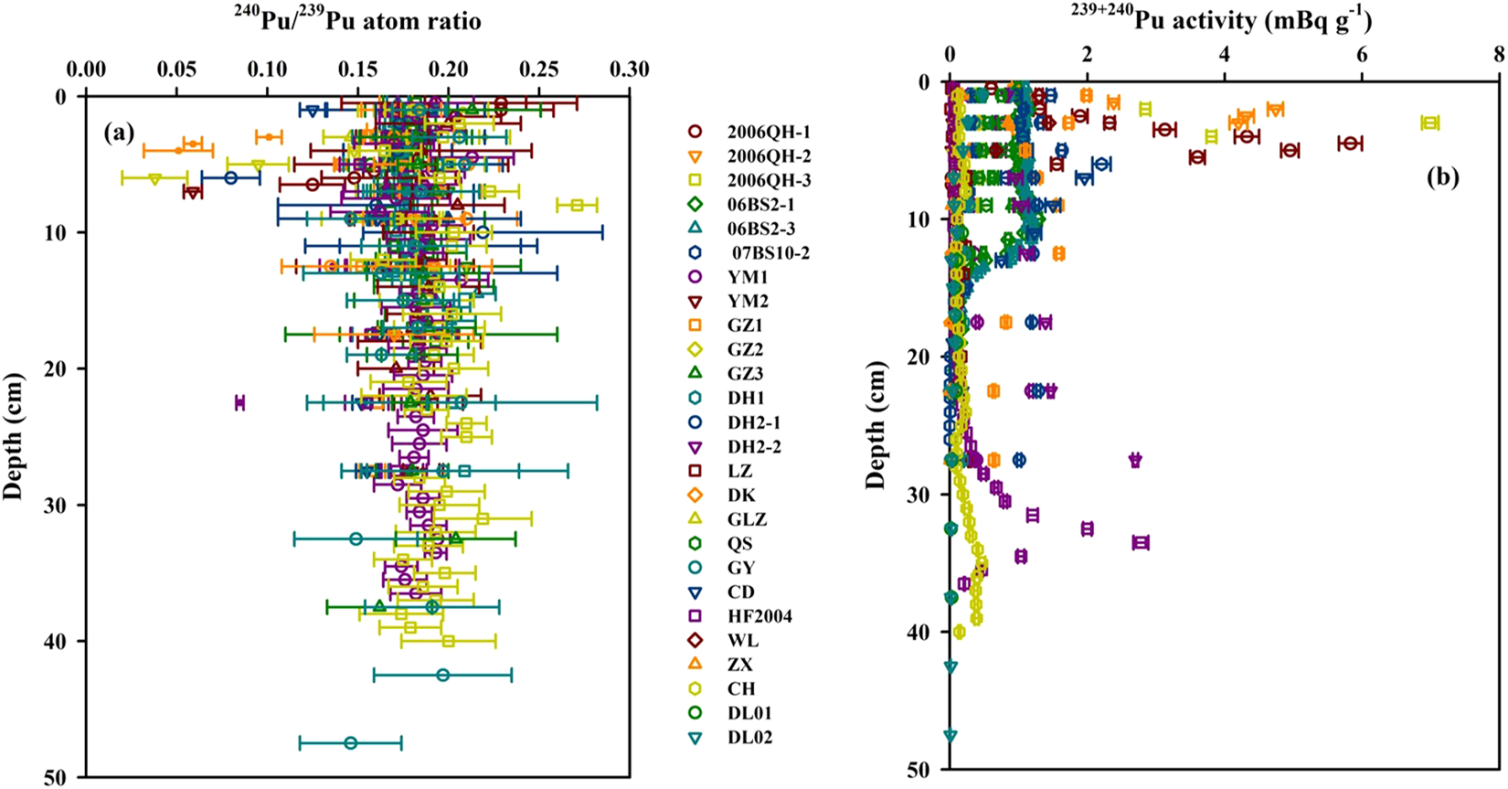
Vertical profiles of ^240^Pu/^239^Pu atom ratio (**a**) and ^239+240^Pu activity (**b**) in soil cores of China. Pu data of profile soils/sediments are cited from the previous studies (2006QH1-3^24^; 06BS2-1, 06BS2-3, 07BS10-2^26^; YM1-2, GZ1-3, DH2-1, DH2-2^32^; LZ^28^; DK, GLZ^37^; QS, CD^35^; GY, ZX, WL^29^; HF2004^33^; CH^34^; DL01-02^39^). This map was prepared with Sigma-Plot professional 10.0 software.

In order to comprehensively investigate the temporal variations of the Pu input and Pu isotopic compositions in the Chinese soils over the past sixty years, I synthesized 26 soil/sediment cores in China, namely, Northwest China: 15 cores^24–26,29,32^, Central China: 2 cores^37^, East China: 1 core^35^, Southwest China: 6 cores^29,33–35^ and Northeast China: 2 cores^39^. The profiles of ^240^Pu/^239^Pu atom ratio and^239+240^Pu activity in the Chinese sediment/soil cores are shown in Fig. 5.

The ^240^Pu/^239^Pu atom ratios in those sediment/soil cores ranged from 0.038 to 0.273 (average = 0.178 ± 0.025, n = 343), with a wide range. As shown in Fig. 5a, two notable features have been observed: 1) the ^240^Pu/^239^Pu atom ratios in the entire soil cores correspond well with the range of global fallout, indicating the dominated Pu source was from the global fallout in the Chinese soils at the past decades. Furthermore, the frequency distribution of ^240^Pu/^239^Pu atom ratios in the Chinese profile samples also showed a typical Gaussian distribution. Among the 343 soil samples, the ranges of 0.17–0.18 and 0.18–0.19 account for 23% and 28%, respectively. About 77% of ^240^Pu/^239^Pu atom ratios correspond well to the global fallout, further suggesting the Pu was mainly sourced from the global fallout in the last sixty years. 2) the lower ^240^Pu/^239^Pu atom ratios observed in the subsurface soils indicated the Pu signature from Lop Nor as suggested by Wu *et al*.^25^, Liao *et al*.^26^ and Bu *et al*.^32^. They also calculated the Pu contribution of Lop Nor to be 20–70%, showing high uncertainty due to be absent of Pu source term value and the accurately estimated method.

The ^239+240^Pu activities in the sediment/soil cores ranged from 0.005 to 6.993 mBq g^−1^, with an average of 0.659 ± 0.889 mBq g^−1^ (n = 343). As shown in Fig. 5b, three features should be noted: (1) the ^239+240^Pu activities showed an exponential decline with an increase of soil depth, indicating there was dramatic influence by the radioactive fallout from the atmospheric nuclear tests since 1940s. (2) the maximum value of ^239+240^Pu activities appeared at the subsurface was indicative of the maximum Pu deposition of global fallout in 1963 because the large-scale nuclear tests were conducted during the period 1961–1962^53^. (3) in the same latitudinal zone, the ^239+240^Pu activities in sediments of Lake were significantly higher than those obtained in the soils, indicating the different migration behaviors in between. The mobility of Pu in the lake sediments was controlled by the changes of pH and oxidation state^54–56^. In contrast, the remigration of Pu in soils was mainly influenced by the Pu partitioning to colloidal and particulate matter^57,58^.

#### ^239+240^Pu inventory in Chinese soils

**Figure 6:**
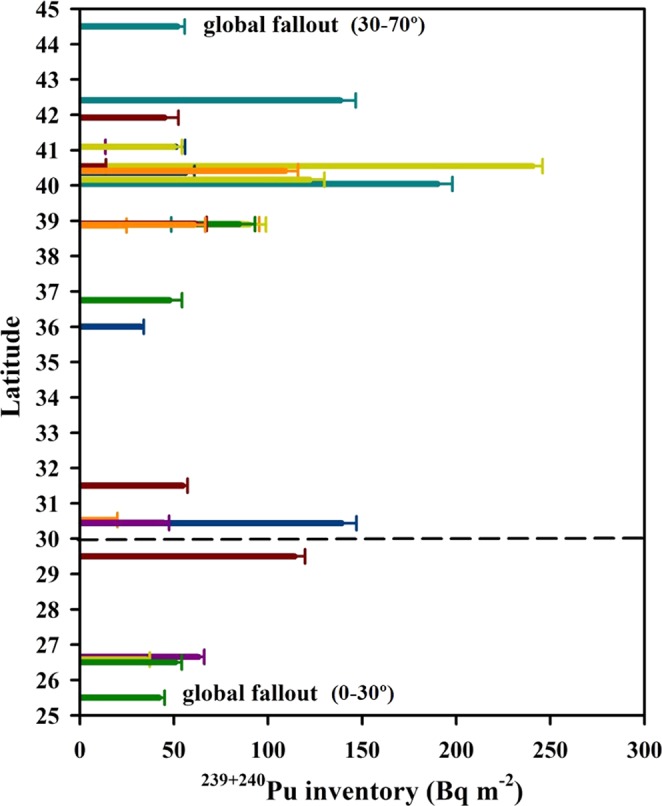
Latitudinal distribution of ^239+240^Pu inventory in Chinese sediment/soil cores. Pu data sourced as same as Fig. 5. This map was prepared with Sigma-Plot professional 10.0 software.

The ^239+240^Pu inventory in the core is a useful indicator for evaluating the sedimentation and accumulation processes and it aids in deciphering the source function, deposition, soil erosion and lateral dispersion of Pu^9^. In this study, ^239+240^Pu inventories in dry soils were calculated by summing their respective activities at each layer, according to the following equation^59^:

1 $$I=\sum _{i=1}^{N}{\rho }_{s}{X}_{i}{A}_{i}$$

where *I* represents the inventories of ^239+240^Pu in the dry soils (mBq cm^−2^), *N* is the number of sampling layers, *ρ*_*s*_ is the solid phase dry density, *X* is the thickness of the sampling interval *i* (cm), and *A* is the activity of the sampled interval (Bq kg^−1^). Uncertainties on inventories are the sum of the propagated error determined for each of the sampling intervals. Combined the ^239+240^Pu inventories in Chinese sediments/soils, the latitudinal distribution of ^239+240^Pu inventory in Chinese sediment/soil cores is presented in Fig. 6. In the latitudinal zone of 20°–30°N, the ^239+240^Pu inventories in soils were slightly higher than the integrated global fallout of 36 Bq m^−2^ published by UNSCEAR^8^. In the latitudinal zone of 30°–45°N, the ^239+240^Pu inventories in sediments/soils were higher than that of global fallout. The high accumulation of Pu in Chinese sediments/soils was potentially caused by the input of Pu signature from Lop Nor in 1964–1980, particularly in the high latitude close to the site of Lop Nor. However, the ^239+240^Pu inventories in Chinese sediment/soil cores were lower than those observed in China Sea (South China Sea^9^: 365.6 ± 3.0 Bq m^−2^; East China Sea^38,60^: 333–407 Bq m^−2^). The latter source received Pu from Pacific Proving Grounds continuously transported into the China Sea via North Equatorial Current and Kuroshio current^9,10^. It is noted that ^239+240^Pu inventories in lake sediments were significantly higher than those obtained in soils in the same latitudinal zone. In general, Pu in anaerobic sediments increases with increasing depth where plenty of organic matter has continuously been supplied, and Pu is likely to disperse and migrate over the whole core because of low *K*_*d*_ values in the strongly anaerobic environment^51^.

### Implication of Pu in the YRSA

The frozen soil, closely related to the terrestrial ecosystem, accumulates the man-made radionuclides originating from the atmospheric nuclear tests and/or the nuclear facilities. Among these radionuclides, Pu is considered as a very radiotoxic nuclide^61^. Pu behavior in soil is a matter of concern in the view of environmental radioactivity because of their high toxicity, long half-lives and a large risk for internal radiation exposure. It is thus important to understand the Pu isotopic composition and source in the YRSA in order to further evaluate their environmental risk. In this study, both ^240^Pu/^239^Pu atom ratio and ^239+240^Pu activity have all pointed towards the Pu source was mainly from the global fallout at present. The activity level of ^239+240^Pu in the YRSA was now between in low values or close to background of global fallout, which does far not cause a Pu toxicity to the downstream drinking water even the frozen soil begins to melt and release Pu to the Yellow River. However, the high deposition and accumulation of Pu was observed in the Chinese sediment/soil cores since the Chinese nuclear tests conducted in the period of 1964–1980, particularly in the downwind of Lop Nor. Note that, the deteriorated soil erosion and associated sedimentation arising from human activities have become a prominent problem, which would potentially result in downstream sedimentation in fields, land degradation, floodplains and water bodies, consequently affect the safety of water quality^39^. Meanwhile, Pu in the environment is serious in two exposure pathways, namely, the Pu in the soils released from the resuspension for inhalation and plant uptake for ingestion should be considered. It is therefore necessary to further monitor the Pu activity levels in the YRSA soil column to ensure the safety of downstream drinking water.

## Conclusion

The ^239+240^Pu activities (0.053–0.836 mBq g^−1^) of surface frozen soils in the YRSA are comparable to those obtained in China elsewhere (0.005–1.990 mBq g^−1^). The ^240^Pu/^239^Pu atom ratios of surface soils in the YRSA, ranging from 0.168 to 0.201 (average = 0.187 ± 0.012, n = 6), are in good agreement with the global fallout of 0.180 ± 0.014^12^. The latitudinal and horizontal distribution patterns of both Pu activity and the isotopic ratio have all pointed towards the YRSA receiving Pu from the global fallout up to now. The activity levels of Pu in the YRSA do far not cause a Pu toxicity to the downstream drinking water even the frozen soil begins to melt and release Pu to the Yellow River. Since the close-in fallout from Lop Nor where the Chinese nuclear tests were carried out during the period of 1964–1980, the high deposition and accumulation of Pu was observed in the Chinese sediment/soil cores through synthesizing the previously published Pu dataset. Finally, I firstly synthesized an expanded dataset from the activity level and atom ratio of Pu isotopes in the Chinese soils and have established the baseline for future environmental risk assessment.

## Materials and Methods

### Sample collection

**Figure 7:**
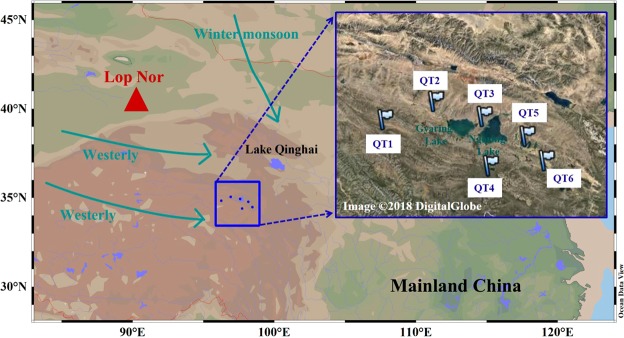
Map showing the sampling sites of frozen soil. Red triangle represents the location of the Chinese nuclear tests in Lop Nor over the period of 1964–1980. Blue rectangle shows the sampling region. Cadmium green arrows denote the directions of Westerly and East Asian winter monsoon^64^. The photograph imagery is obtained from http://www.google.com/maps (Map data: Google, DigitalGlobe). This map was drawn using a free software Ocean Data View (ODV 5.1.2) (Schlitzer, R., Ocean Data View, odv.awi.de, 2018).

Six surface soil samples were collected in the YRSA (close to Ngoring Lake and Gyaring Lake), which is about 800–1000 km southeast of Lop Nor, the Chinese nuclear test site during the period of 2014. Detailed sampling locations are marked in Fig. 7. Longitudes, latitudes and sampling date at the stations are presented in Table S1. After collection, they were dried at 110 °C for 24 h and pulverized using agate mortar and pestle sets. Then, they were calcined in a muffle furnace at 500 °C for 6 h to decompose the organic matter in preparation for Pu isotope analysis.

### Pu isotope analysis

The separation of Pu has been used the two–stage ion–exchange chromatography. Briefly, soil samples (~5.0 g) were weighed out and a known amount of ^242^Pu (IRMM–085, European Commission Joint Research Centre, Belgium) was added to the soil samples as a yield monitor. The spiked samples were digested by heating on a hot-plate at 180–200 °C for ~4 h using 30 mL concentrated (conc.) HNO_3_ in a sealed Teflon digestion tube. Pu in the sample solution was subsequently purified by the two-stage anion-exchange columns using AG 1–X8 and AG MP–1M (Bio–Rad). A small drop of the final sample solution was dissolved in 4% ultrapure HNO_3_ (1.0 mL) and filtered for MC–ICP–MS analysis. The determination of Pu isotopes was conducted using MC–ICP–MS (Nu plasma HR, Nu Instruments Ltd., England) in a low resolution mode in order to obtain the maximal sensitivity in Xiamen University. The DSN–100 high efficiency sample introduction system with a membrane desolvation unit and a conical concentric nebulizer was used. The flow chart of analytical procedure for the Pu isotopes in soil/sediment samples are presented in Fig. S2. The chemical yield for Pu resulting from this analytical procedure was 66.4% ± 5.6%. In addition, for Pu measurements with MC–ICP–MS, the most significant interferences are usually caused by the formation of isobaric uranium hydrides (^238^UH^+^) and peak tailing from the ^238^U^+^ peak, resulting in overestimation of the ^239^Pu signal. Our analytical procedure employed in this work was able to effectively eliminate the U interferences by achieving an extremely high U decontamination factor of 6.0 × 10^7^, which was comparable to previously reported values (3.0 × 10^7^–1.0 × 10^8^)^62^.

The data quality and the mass bias correction were assured by regular analyses of the IAEA–443 (Irish Seawater) certified reference material (^240^Pu/^239^Pu = 0.228 ± 0.005, n = 2, verified value: 0.229 ± 0.006). The analytical method was also validated by analyzing with other reference materials: IAEA–384 (Fangataufa Lagoon Sediment) and IAEA–385 (Irish Sea Sediment) (International Atomic Energy Agency). The accuracies of the ^239+240^Pu activities and the ^240^Pu/^239^Pu atom ratios were in good agreement with the certified and previously reported values (Table S2). In addition, the operational blank count rates for ^239^Pu and ^240^Pu were analyzed following the same chemical procedure for Pu determination in soil. The limit of detection (LOD) was calculated based on the International Union of Pure and Applied Chemistry recommendations^63^. The LOD was calculated to be 0.44 fg mL^−1^ for ^239^Pu and 0.36 fg mL^−1^ for ^240^Pu.

## Supplementary information

Supplementary Information
