# Supplementary material for: Isotopic composition and source of plutonium in the Qinghai-Tibet Plateau frozen soils
Source: Sci Rep. 2019 May 27;9:7861. doi: 10.1038/s41598-019-44391-0 (PMC6536505; doi:10.1038/s41598-019-44391-0)
Supplement: Supplementary file 1 — Supplementary Information [file 41598_2019_44391_MOESM1_ESM.docx]

**Supplementary information**

**Isotopic composition and source of plutonium in the Qinghai-Tibet Plateau frozen soils**

Junwen Wu^a*,b^

^a^ Institute of Marine Biology, College of Science, Shantou University, Shantou 515063, China

^b^ State Key Laboratory of Marine Environmental Science, Xiamen University, Xiamen 361102, China

*** Corresponding author:**

E-mail: [wujw@stu.edu.cn](mailto:wujw@stu.edu.cn)

**This supplementary information contains two figures, two tables and references.**

**(1) Figure S1.** The yield of Chinese atmospheric nuclear tests at the site of Lop Nor during the period of 1964-1980.

**(2) Figure S2.** Schematic diagram of the analytical procedure for Pu isotopes in soil.

**(3) Table S1.** ^239+240^Pu activities and ^240^Pu/^239^Pu atom ratios in surface soils of the Yellow River source area.

**(4) Table S2.** Analytical results of ^240^Pu/^239^Pu atom ratios and ^239+240^Pu activities in the reference materials

**(5) References**


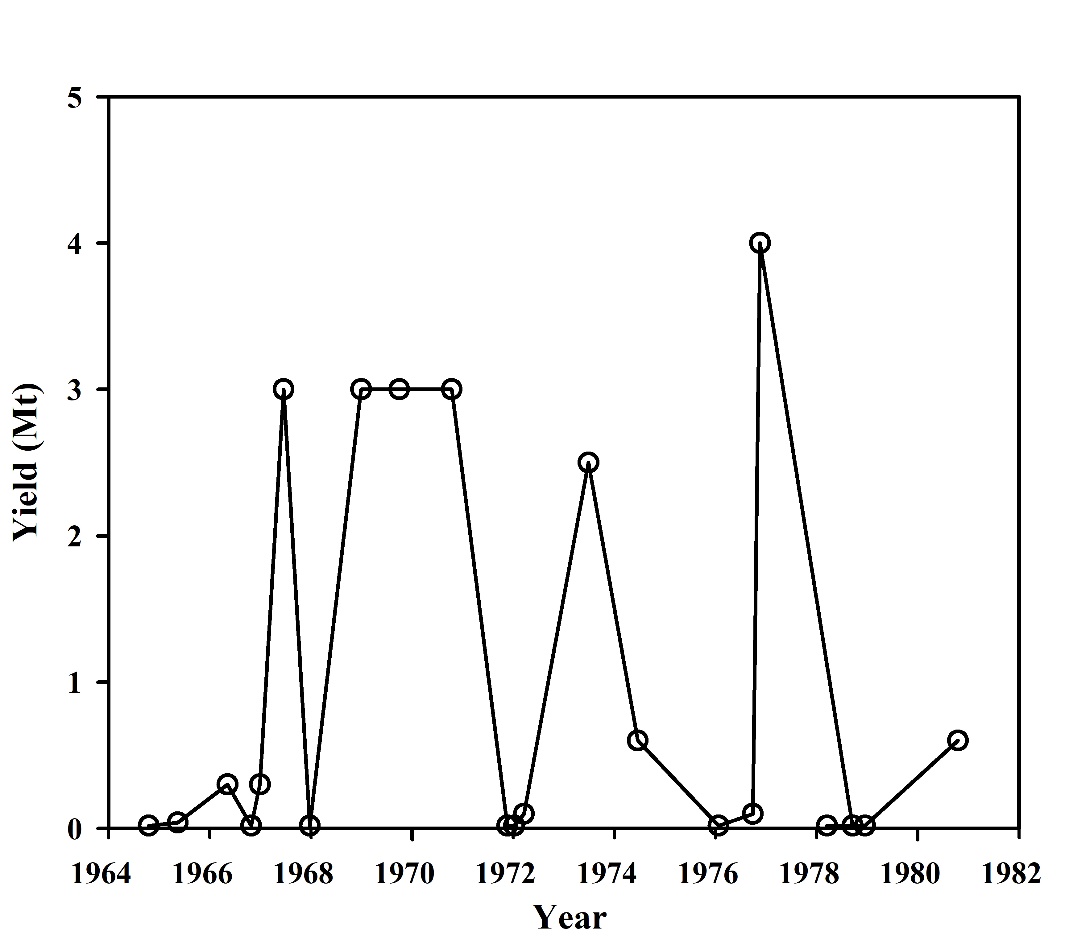


**Figure S1.** The yield of Chinese atmospheric nuclear tests at the site of Lop Nor during the period of 1964-1980 (data source from UNSCEAR^1^).

**
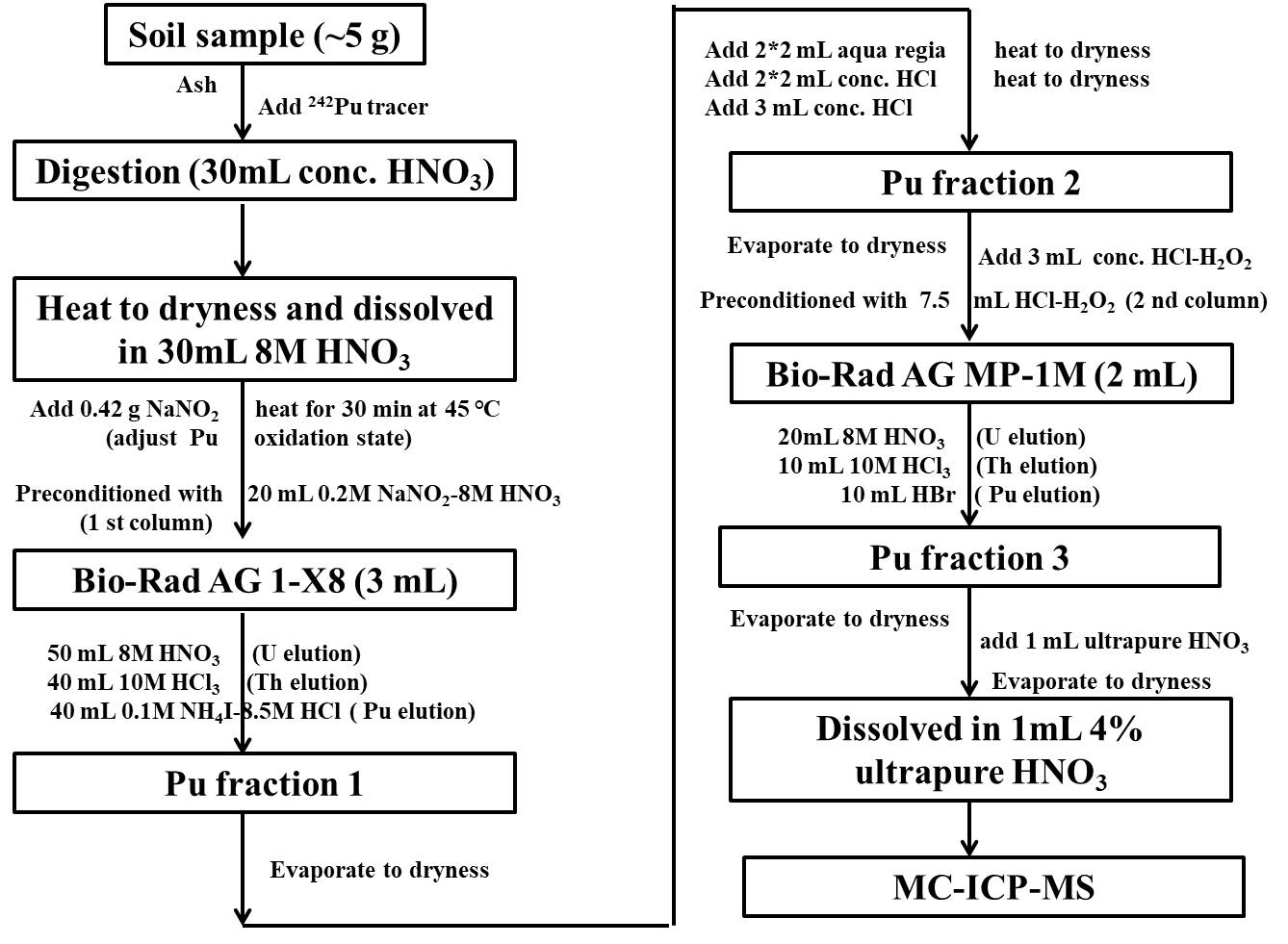
**

**Figure S2.** Schematic diagram of the analytical procedure for Pu isotopes in soil.

**Table S1.** ^239+240^Pu activities and ^240^Pu/^239^Pu atom ratios in surface soils of the Yellow River source area

| Station | Latitude(°N) | Longitude (°E) | Sampling date | texture | Layer (cm) | ^239+240^Pu activities (mBq g^-1^) | ^240^Pu/^239^Pu atom ratio |
| --- | --- | --- | --- | --- | --- | --- | --- |
| QT1 | 34.845 | 96.249 | 2014-04-20 | clay | 0-2 | 0.229±0.003 | 0.184±0.004 |
| QT2 | 35.064 | 96.914 | 2014-04-20 | silt | 0-2 | 0.053±0.002 | 0.201±0.005 |
| QT3 | 34.943 | 97.588 | 2014-04-25 | silty clayey | 0-2 | 0.553±0.013 | 0.191±0.003 |
| QT4 | 34.411 | 97.732 | 2014-04-23 | silty clayey | 0-2 | 0.836±0.008 | 0.168±0.003 |
| QT5 | 34.781 | 98.162 | 2014-04-27 | clay | 0-2 | 0.160±0.003 | 0.181±0.004 |
| QT6 | 34.487 | 98.455 | 2014-04-24 | silty clayey | 0-2 | 0.415±0.008 | 0.196±0.004 |

**Table S2.** Analytical results of ^240^Pu/^239^Pu atom ratios and ^239+240^Pu activities in the reference materials

| Reference materials | Measured ^239+240^Pu activity (mBq) | Added ^239+240^Pu activity (mBq ) | Measured ^240^Pu/^239^Pu  atom ratio | ^240^Pu/^239^Pu Information values | Accuracy^(a)^ | Precision | Reference |
| --- | --- | --- | --- | --- | --- | --- | --- |
| IAEA-443 | 2.96±0.03  (n=2) | 2.90-3.04 | 0.228±0.005  (n=2) | 0.229±0.006 | 0.3% (activity)  0.4% (ratio) | 1.0% (activity)  2.2% (ratio) | 2 |
| Reference materials | Measured ^239+240^Pu activity (mBq g^-1^) | Certified ^239+240^Pu activity (mBq g^-1^) | Measured ^240^Pu/^239^Pu  atom ratio | ^240^Pu/^239^Pu  literature values | Accuracy | Precision | Reference |
| IAEA-384 | 108.12±3.08  (n=6) | 107  (103-110) | 0.050±0.001  (n=6) | 0.045-0.053  (n=9) | 1.0% (activity) | 2.8% (activity)  2.0% (ratio) | 3-6 |
| IAEA-385 | 2.93±0.03  (n=6) | 2.96  (2.89-3.00) | 0.185±0.003  (n=6) | 0.178-0.192  (n=3) | 1.0% (activity) | 1.0% (activity)  1.6% (ratio) | 4, 7 |

^(a)^ The percentage indicates the precision and accuracy of the ^240^Pu/^239^Pu atom ratio and^239+240^Pu activity, respectively.

Uncertainties of the data are calculated based on ±1σ counting statistics and standard propagation of errors.

**References**

1. UNSCEAR Report: Sources and effects of ionizing radiation. United Nations scientific committee on the effects of atomic radiation exposures to the public from man-made sources of radiation; United Nations, New York, (2000).
2. Pham, M.K., Betti, M., Povinec, P.P., Benmansour, M., Bunger, V., Drefvelin, J., Engeler, C., Flemal, J.M., Gasco, C., Guillevic, J., Gurriaran, R., Groening, M., Happel, J.D., Herrmann, J., Klemola, S., Kloster, M., Kanisch, G., Leonard, K., Long, S., Nielsen, S., Oh, J.S., Rieth, P.U., Ostergren, I., Pettersson, H., Pinhao, N., Pujol, L., Sato, K., Schikowski, J., Varga, Z., Vartti, V.P., Zheng, J. A certified reference material for radionuclides in the water sample from Irish Sea (IAEA-443). *J. Radioanal. Nucl. Chem.* 288, 603–611 (2011).
3. Povinec, P.P., Pham, K.K., Sanchez-Cabeza, J.A., Barci-Funel, G., Bojanowski, R., Boshkova, T., Burnett, W.C., Carvalho, F., Chapeyron, B., Cunha, I.L., Dahlgaard, H., Galabov, N., Fifield, L.K., Gastaud, J., Geering, J.J., Gomez, I.F., Green, N., Hamilton, T., Ibanez, F.L., Ibn-Majah, M., Kanish, G., Kenna, T.C., Kloster, M., Korun, M., Liong-Wee-Kwong, L., La-Rosa, J.J., Lee, S.H., Levy-Palomo, I., Malatova, M., Maruo, Y., Mitchell, P., Murciano, I.V., Nelson, R., Nouredine, A., Oh, J.S., Oregioni, B., Le-Petit, G., Petterson, H.B.L., Reineking, A., Smedley, P.A., Suckow, A., Van-der-Struijs, T.D.B., Voors, P.I., Yoshimizu, K., Wyse, E. Reference material for radionuclides in sediment IAEA-384 (Fangataufa Lagoon sediment). *J. Radioanal. Nucl. Chem.* 273, 383–393 (2007).
4. Lindahl, P., Keith-Roach, M., Worsfold, P., Choi, M.S., Shin, H.S., Lee, S.H. Ultra-trace determination of plutonium in marine samples using multi-collector inductively coupled plasma mass spectrometry. *Anal. Chim. Acta* 671, 61–69 (2010).
5. Lindahl, P., Worsfold, P., Keith-Roach, M., Andersen, M.B., Kershaw, P., Leonard, K., Choi, M.S., Boust, D., Lesueur, P. Temporal record of Pu isotopes in inter-tidal sediments from the northeastern Irish Sea. *Sci. Total Environ.* 409, 5020–5025 (2011).
6. Godoy, M.L.D.P., Godoy, J.M., Roldao, L.A., Tauhata, L. Determination of total content and isotopic compositions of plutonium and uranium in environmental samples for safeguards purposes by ICP-MS. *J. Environ. Radioact.* 100, 613–625 (2009).
7. Pham, M.K., Sanchez, J.A., Povinec, P.P., Andor, K., Arnold, D., Benmansour, M., Bikit, I., Carvalho, F.P., Dimitrova, K., Edrev, Z.H., Engeler, C., Fouche, F.J., Garcia-Orellana, J., Gasco, C., Gastaud, J., Gudelis, A., Hancock, G., Holm, E., Legarda, F., Ikaheimonen, T.K., Ilchmann, C., Jenkinson, A.V., Kanisch, G., Kis-Benedek, G., Kleinschmidt, R., Koukouliou, V., Kuhar, B., LaRosa, J., Lee, S.H., LePetit, G., Levy-Palomo, I., Liong-Wee-Kwong, L., Llaurado, M., Maringer, F.J., Meyer, M., Michalik, B., Michel, H., Nies, H., Nour, S., Oh, J.S., Oregioni, B., Palomares, J., Pantelic, G., Pfitzner, J., Pilvio, R., Puskeiler, L., Satake, H., Schikowski, J., Vitorovic, G., Woodhead, D., Wyse, E. A new certified reference material for radionuclides in Irish Sea sediment (IAEA–385). *Appl. Radiat. Isoto.* 66, 1711–1717 (2008).
